# A Microfluidic Love-Wave Biosensing Device for PSA Detection Based on an Aptamer Beacon Probe

**DOI:** 10.3390/s150613839

**Published:** 2015-06-11

**Authors:** Feng Zhang, Shuangming Li, Kang Cao, Pengjuan Wang, Yan Su, Xinhua Zhu, Ying Wan

**Affiliations:** 1 School of Mechanical Engineering, Nanjing University of Science and Technology, Nanjing 210094, China; E-Mails: zhangfeng_njust@163.com (F.Z.); lishuangming1989@163.com (S.L.); caokang666@163.com (K.C.); suyan@njust.edu.cn (Y.S.); 2 School of Environmental and Biological Engineering, Nanjing University of Science and Technology, Nanjing 210094, China; E-Mail: wpjshirley@163.com

**Keywords:** Love wave, biosensing, PSA, aptamer, stem-loop probe, microfluidic, PDMS

## Abstract

A label-free and selective aptamer beacon-based Love-wave biosensing device was developed for prostate specific antigen (PSA) detection. The device consists of the following parts: LiTaO_3_ substrate with SiO_2_ film as wave guide layer, two set of inter-digital transducers (IDT), gold film for immobilization of the biorecongniton layer and a polydimethylsiloxane (PDMS) microfluidic channels. DNA aptamer, or “artificial antibody”, was used as the specific biorecognition probe for PSA capture. Some nucleotides were added to the 3'-end of the aptamer to form a duplex with the 3'-end, turning the aptamer into an aptamer-beacon. Taking advantage of the selective target-induced assembly changes arising from the “aptamer beacon”, highly selective and specific detection of PSA was achieved. Furthermore, PDMS microfluidic channels were designed and fabricated to realize automated quantitative sample injection. After optimization of the experimental conditions, the established device showed good performance for PSA detection between 10 ng/mL to 1 μg/mL, with a detection limit of 10 ng/mL. The proposed sensor might be a promising alternative for point of care diagnostics.

## 1. Introduction

As known, early diagnosis of cancer can greatly improve the survival chances of patients. However, due to the fact that there are trace protein biomarkers in serum, it is difficult to accurately detect protein biomarkers [1]. Numerous technologies have been developed for protein marker detection, such as the enzyme-linked immunosorbent assay (ELISA) [2], radioimmunoassay [3], fluorescence immunoassay [4], electrophoretic immunoassay [5], mass spectrometric immunoassay [6], immune-polymerase chain reaction (PCR) assay [7], and immune-rolling circle amplication (RCA) assay [8]. Though some of these technologies are widely used, they still generally have some disadvantages such as being time consuming, labor consuming and requiring expensive instruments. Thus, considerable efforts have been made to develop operationally simple, ultrasensitive and easily automated devices for cancer biomarker diagnostics [9,10,11,12].

Surface acoustic wave (SAW) biosensors, with their inherent advantages of high sensitivity, low cost, low power requirement and real-time monitoring, have been applied in clinical diagnosis [13,14,15]. Love-wave sensors are a special type of surface acoustic wave (SAW) sensors which use shear horizontal waves guided in a layer on the surface of the sensor to reduce energy dissipation of the acoustic wave into the fluid and to increase the surface sensitivity. Therefore, they possess sufficient sensitivity to detect mass loadings in liquids as low as 1.0–2.0 ng/cm^2^ [16]. Besides, there are some other demands for a point-of-care diagnostic system. On the way towards automated detection and real-time monitoring, microfluidic SAW sensors have been developed with about 4–5 times higher sensitivity compared with quartz crystal microbalances [17].

The biorecognition elements play an important role in any immunosensor. Several Love-wave immunosensors have been developed for protein biomarker detection by using antibodies as biorecogniton elements [18,19,20]. However, the immobilization of antibodies often leads to the decrease of reaction activity. ssDNA or RNA aptamers, which are called “artificial antibodies” [21,22,23], thus represent promising alternatives as molecule recognition elements in bioassays, due to their inherent advantages of sensitivity, selectivity, stability and easy with colorimetric [24,25], fluorescent [26,27], quartz crystal microbalance [28], electrochemical systems [29,30], and so on.

Schlensog *et al.*, reported a Love-wave biosensor prepared by immobilizing a single strand DNA (ssDNA) aptamer as ligand [31]. Human thrombin was thus detected with a detection limit of approximately 72 pg/cm^2^. Other analytes were also detected with high sensitivity and specificity, such as HIV-1 Rev peptide (77 pg/cm^2^) and the complementary strand DNA.

Here, we report a microfluidic Love-wave biosensor for real-time PSA detection. In order to realize automated quantitative sample injections, a microfluidic channel was designed and fabricated from poly(dimethylsiloxane) (PDMS). For specific capture of PSA, a DNA aptamer-beacon with a “stem-loop” structure was used as a specific biorecognition probe. Taking advantage of the selective target-induced assembly changes that arise from the “aptamer beacon”, highly selective and specific detection of PSA could be achieved.

## 2. Experimental Section

### 2.1. Fabrication of the Two-Channel Delay-Line Sensor Device

**Figure 1:**
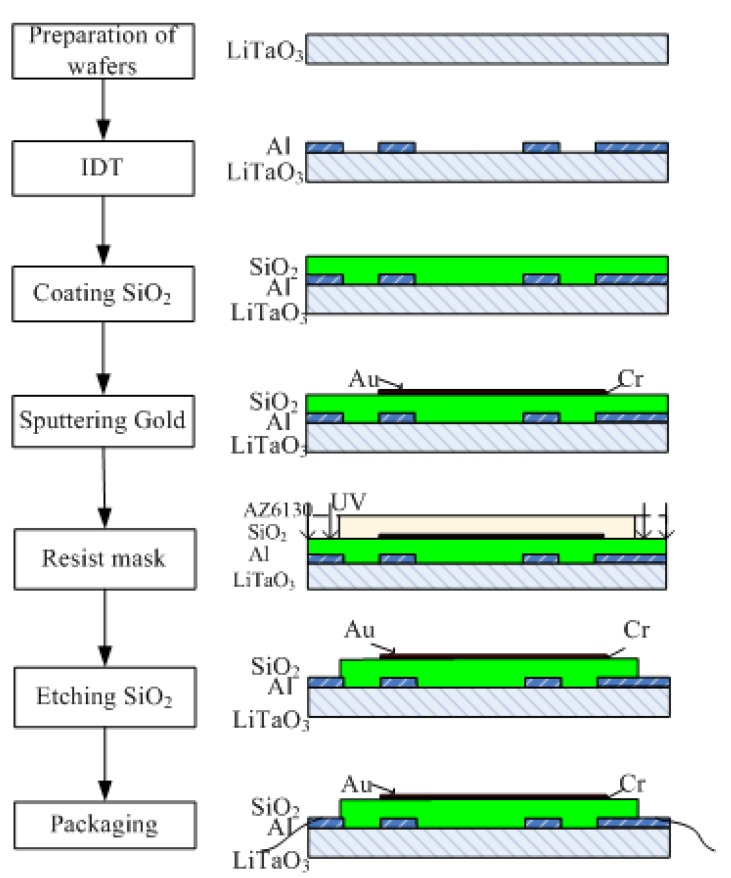
Fabrication of the Love-wave sensor device.

The sensor chips were prepared by lithographic deposition on lithium tantalate wafers (36°, y-cut, x-propagating LiTaO_3_, Figure 1). Briefly, a metallization step was performed on cleaned LiTaO_3_ with 1500 Å aluminum using an electron-beam evaporator. Then, wafers were patterned with four inter-digital transducers (IDT) patterns with sets of transducers and delay lines using SPR6112B positive tone photoresist. The aluminum was etched in acid, followed by rinsing with acetone, isopropyl alcohol, and deionized water. Next, silicon dioxide (SiO_2_) layers of different thicknesses (0, 1, 2, 4 μm) were deposited as a film on the wafer using lift-off plasma enhanced chemical vapor deposition. In order to improve the SiO_2_ quality, the deposition rate was set at 250 nm/min [32]. During the deposition, a 3 min interruption after 5 min deposition was used to release the strain/stress between the SiO_2_ layer and LiTaO_3_. Then, a 30 nm chromium film and a 50 nm gold film were deposited by a magnetron sputtering process via an AZ5214 resist mask. Afterward, wafers were pretreated with hexamethylisiloxane (HMDS) in a vacuum oven, and then etched with reactive ion etching (RIE) to access the electrical contact pads via a positive photoresist AZ6130 mask. Finally, unwanted parts were removed by rinsing in acetone, methanol, and isopropanol.

The parameters of the fabricated device was as follows: two sets of transmitting and receiving IDTs were placed at each end and the center-to-center distance between them was 10 mm. The IDTs had an aperture of 3.12 mm and the number of finger pairs was 200. One of the channels had a center frequency of about 197 MHz with a wavelength of 21.17 μm. The other channel had a center frequency of about 198 MHz with a wavelength of 21.28 μm. The propagation areas were covered with a 50 nm gold film.

### 2.2. Fabrication of the PDMS Microfluidic Chip

**Figure 2:**
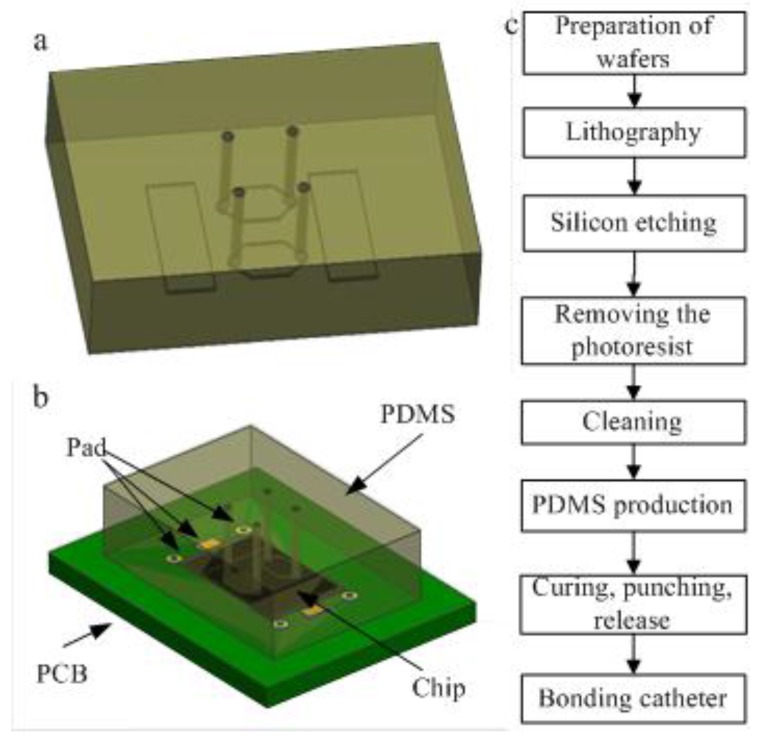
(**a**) Illustration of the PDMS chip; (**b**) Device package diagram; (**c**) Process flow diagram of the PDMS microfluidic chip.

PDMS was used to fabricate the microfluidic chip due to its good biocompatibility. Microfluidic channels were designed as shown in Figure 2a. The middle portion of the cavity was a reaction chamber and the rectangles were designed to cover the IDT part to avoid sound absorption. The structure of the final device package is illustrated in Figure 2b and the process flow diagram is shown in Figure 2c. The first five steps were used to make a silicon template. Then the PDMS chip was made based on the template and finally integrated with the Love-wave chip.

### 2.3. Functionalization of the Sensor

Aptamer with a sequence from the literature [33] was purchased from Sangon Biotech Co. (Shanghai, China). The aptamer immobilization steps are as follows: briefly, the aptamer was immobilized in each channel at a concentration of 1 μM in phosphate-buffered saline (PBS). The sensitive area was immersed in PSA aptamer solution for 4 h at room temperature. PBS was used to remove the unreacted reagent after each step. After that, the chip was incubated for 4 h at room temperature in BSA solution at a concentration of 1% in PBS.

### 2.4. PSA Detection Using the SAW Biosensor

**Figure 3:**
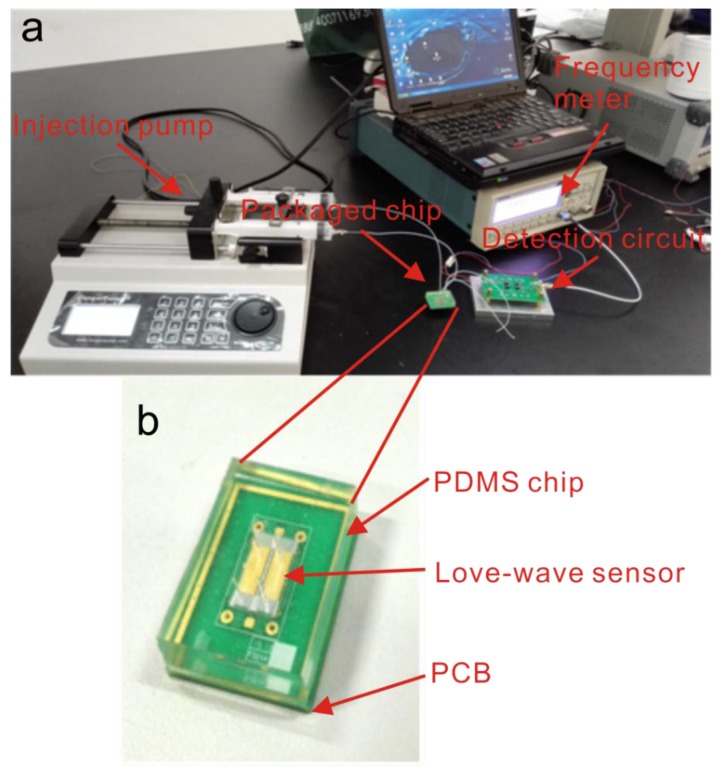
Photograph of (**a**) the biotesting system and (**b**) the Love-wave device.

A photo of the experimental set-up is shown in Figure 3. The biosensing experiments were carried out in a controlled temperature and humidity chamber. A syringe pump was used to control the sample injections. After the functionalization of the Love-wave sensor, two channels were filled with sample and reference solution, respectively. In the biosensing experiment, the sample channel was injected with PSA diluted in 1% BSA (PBS buffer, pH 7.2) and the reference channel contained 1% BSA. In the control experiment, the sample channel was injected with carcinoembryonic antigen (CEA) diluted in 1% BSA and the reference solution was 1% BSA. Then the signals were collected using a FCA3103 Frequency Counter (Tektronix, Beaverton, OR, USA). In order to facilitate observation and data management, a custom acquisition program was developed using Labview (National Instruments, Austin, TX, USA).

## 3. Results and Discussion

### 3.1. Two-Channel Delay-Line Sensor Device

SAW devices show great temperature effects, which restrict their application in biosensing [34]. In order to minimize the temperature effect, a two-channel self-oscillation circuit with a detection channel and a reference channel was designed for the investigation of the frequency-temperature sensitivity. When the self-oscillation conditions are satisfied, the output frequency of the oscillating circuit can be sensitive to the environmental factor changes.

**Figure 4:**
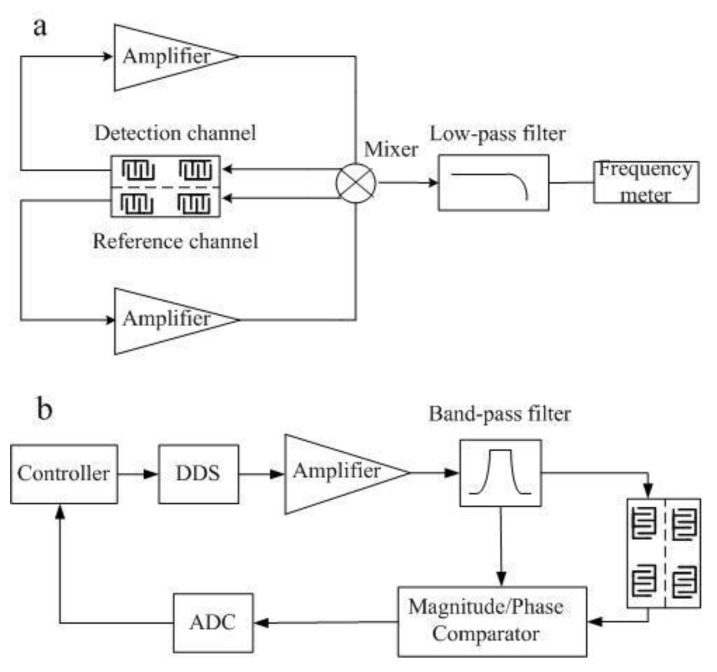
(**a**) Self-excitation oscillation electric circuit; (**b**) Schematic diagram of the SAW oscillator.

As shown in Figure 4a, the detection system consists of an amplifier, a mixer, and low pass filter and frequency meter. A MAX2611 device which has a 3 dB bandwidth of 1100 MHz (Maxim Integrated Products, Inc., San Jose, CA, USA) was selected as the amplifier. A uPC2758TB (Nippon Electric Co., Ltd., Tokyo, Japan) was used as the mixer. A LFCN-400 (Mini-Circuits Co., Ltd., New York, NY, USA) was employed as low pass filter, and a FCA3103 (Tektronix Co., Ltd.) was used as the frequency meter. After frequency mixing and low-pass filtering, the frequency difference signal of two channels was measured. By using this detection system, the effects of temperature were almost eliminated. Since the temperature may affect the quiescent point of the amplifier, the open loop phase would change, resulting in a frequency shift of the SAW oscillator. Figure 4b is the schematic diagram of the Love-wave oscillating circuit. The proposed structure is simpler compared with others that need a Voltage Controlled Oscillator (VCO) or Automatic Gain Controller (AGC). Moreover, this design avoids having the amplifier working in a nonlinear area, which improves the stability of the oscillating circuits. To test the frequency-temperature sensitivity of the Love-wave device, a DS18B20 temperature sensor and a frequency output device were added to the detection system.

Theoretically, the waveguide layer thickness affects the acoustic loss, Love wave velocity, and sensitivity. However, the frequency-temperature efficiency is the key point that affects the sensitivity. As reported by Liu and Flewitt, the best sensitivity can be obtained with a waveguide layer thickness of 0.255λ (where λ is the Love-wave wavelength) [34]. The wavelength used in this work was 21.28 µm and the theoretical best thickness was 5.4 µm.

**Figure 5:**
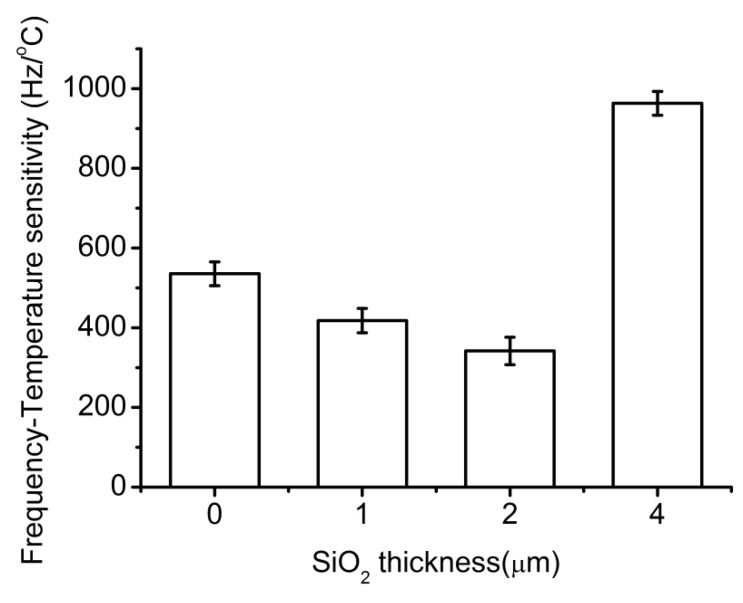
Frequency-temperature sensitivity *vs.* SiO_2_ thickness.

Frequency-temperature sensitivity was investigated for Love-wave devices with different wave guide layer thicknesses. As shown in Figure 5, the frequency-temperature sensitivity first decreased with the increase of waveguide layer thickness, but then increased. Tomar *et al.*, reported that the temperature coefficient of delay (TCD) of the waveguide layer could be as low as zero at a certain thickness when the temperature coefficient of the piezoelectric substrate and the waveguide layer are opposite [35]. The tendency in Figure 5 was consistent with this theory and a thickness of 2 μm was found to be the best. However, 2 μm was not the theoretical best thickness (the theoretical best thickness was 5.4 µm). The reason might be that the fabrication process in this work was not the same as in the literature [34].

### 3.2. Simulation of Velocity Distribution of Fluid in PDMS Channel

**Figure 6:**
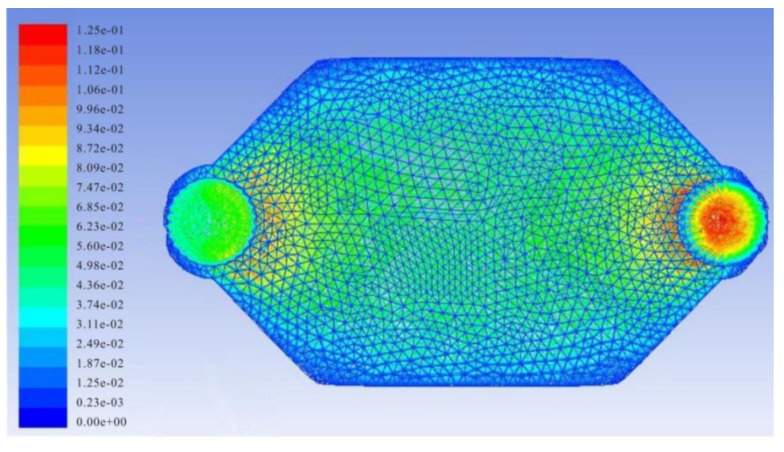
Simulation of velocity distribution of fluid in the reaction chamber.

A PDMS chip was designed to perform automated quantitative sample injections and biorecogniton reactions. The configuration of the reaction chamber was a key factor for reaction efficiency. In order to investigate the velocity distribution of fluid in the reaction chamber, Finite Element Analysis (FEA) was used to simulate the fluidic velocity. (Figure 6) The FEA parameters were as follows: conventional tetrahedral layout was used to mesh the field. The simulation fluid was set as water. The inlet fluid velocity was set to 0.05 m/s. The outlet pressure was set to 0 Pa and the other planes were set as walls. Then calculation was done using ANSYS 13.0 (ANSYS Co., Ltd., Pittsburgh, PA, USA) and a laminar flow model. As shown in Figure 6, the fluidic velocity in the reaction chamber is distributed evenly in the spindle type chamber, which offers mild reaction conditions for the biosensing assay.

### 3.3. Detection of PSA

**Figure 7:**
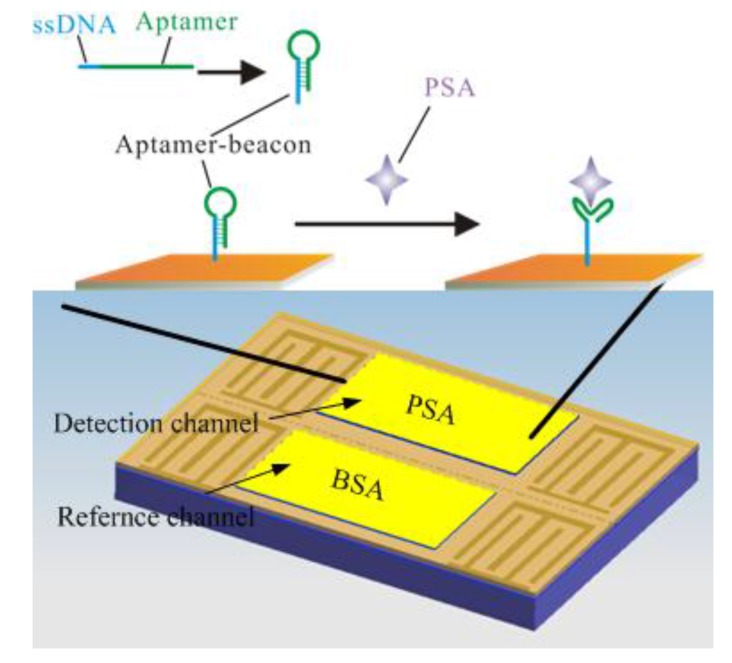
Schematic of the Love-wave immunosensor: a two channel Love-wave sensor chip is used. One channel is for PSA and the other is for the non-specific protein BSA. For specific binding of the target PSA, an aptamer beacon probe was immobilized on the gold film.

The schematic of the Love-wave immunosensor is shown in Figure 7. As is known, the affinity force between a target and an aptamer is due to the specific secondary structure of the aptamer. In the presence of the target, the base paring of the aptamer creates a secondary structure such as a short helical arm to accommodate the target and form an “aptamer-target” complex. In this work, the capture probe has a “stem-loop” structure, which is stable and resist to non-specific binding. While the target protein is added, the “aptamer-target” affinity was higher than that of the aptamer beacon, leading to an adjustment of the probe to accommodate the target. It is reported that the target-induced conformational switching can efficiently reduce the non-specific binding, resulting in an improvement of selectivity [36,37,38].

**Figure 8:**
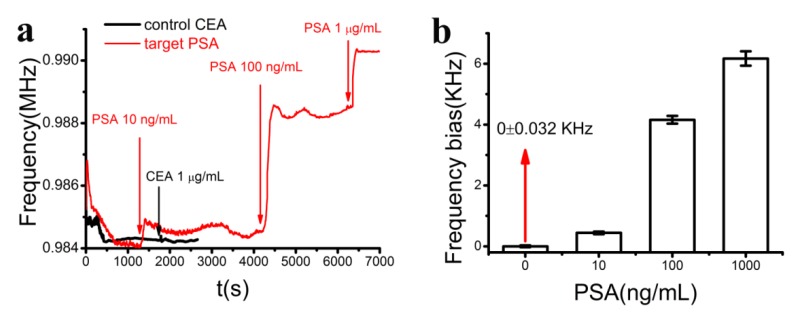
(**a**) The corresponding frequency signals of the sensor during the injection of the target PSA with concentrations of 10 ng/mL, 100 ng/mL and 1 μg/mL (red) and control CEA with the concentration of 1 μg/mL (black); (**b**) Statistical evaluation of averaged frequency bias *vs* PSA concentration. Data were collected from at least three independent sets of experiments.

The frequency signals were collected during the injection of the target PSA in the range of 10 ng/mL–1 μg/mL and control CEA solutions with a concentration of 1 μg/mL (Figure 8a). The frequency signals dropped at the beginning due to the temperature drift induced by the electrical connection. After a few minutes (0–600 s), the signal reached saturation, indicating the stability of the sensor. When the target protein was added, the signal increased fast and reached a saturation point within 100 s. The frequency bias was increased with PSA concentrations. When the control protein CEA was added, no signal changes could be observed, indicating the good selectivity of this sensor. The frequency biases were evaluated with respect to PSA concentration (Figure 8b). The background signal was 0 ± 0.032 KHz, which means that the detection limit of this Love-wave biosensor was lower than 10 ng/mL. The detection limit was not low enough for real sample detection, however, it can be predicted that high sensitivity would be obtained by using appropriate signal amplification methods such as nanomaterial-based technologies.

## 4. Conclusions/Outlook

PSA is a specific biomarker for prostate cancer, which is widely used in prostate cancer diagnosis. We have demonstrated a real-time, label-free sensing system for the rapid detection of PSA. A microfluidic Love-wave sensor was employed to realize real-time monitoring and automated quantitative sample injection. To improve the selectivity, an aptamer beacon was used to resist the non-specific binding. By using this system, a detection limit of 10 ng/mL was obtained. Further work will focus on real-time monitoring and point-of-care use. The Love-wave biosensors can be integrated with wireless electronic readers connected to a smartphone, resulting in a simple positive/negative result for field workers on the front lines of early cancer diagnosis.
